# T cells upon activation promote endothelin 1 production in monocytes via IFN-γ and TNF-α

**DOI:** 10.1038/s41598-017-14202-5

**Published:** 2017-11-03

**Authors:** Shoshi Shinagawa, Takahiro Okazaki, Mari Ikeda, Kazuo Yudoh, Yaz Y. Kisanuki, Masashi Yanagisawa, Kimito Kawahata, Shoichi Ozaki

**Affiliations:** 1 Division of Rheumatology and Allergology, Department of Internal Medicine, St. Marianna University School of Medicine, Kawasaki, Japan; 2 Department of Frontier Medicine, Institute of Medical Science, St. Marianna University School of Medicine, Kawasaki, Japan; 3 Department of Neurology, Division of Neurogenetics, The Ohio State University Wexner Medical Center, Columbus, Ohio USA; 4 International Institute for Integrative Sleep Medicine (WPI-IIIS), University of Tsukuba, Tsukuba, Japan

## Abstract

Endothelin 1 (ET-1), mainly produced from vascular endothelial cells, induces vasoconstriction in physiological conditions. The endothelin receptor antagonist is among the most effective agents for pulmonary hypertension. However, little is known about the production source of ET-1 in inflammation and immunity. Here, we studied whether T cell-mediated ET-1 production system exists and operates independent of the production system in vascular endothelial cells. ET-1 production was readily detectable in the culture supernatant of human PBMCs and murine spleen cells stimulated with anti-CD3 antibody. Immunocytostaining showed that ET-1-producing cells emerged only in PBMCs stimulated with anti-CD3 antibody. Using the Transwell system, both murine and human monocytes sorted with magnetic beads in the inner chamber produced ET-1 when T cells were activated with antigen or anti-CD3 antibody in the outer chamber. This ET-1 production was inhibited by anti-IFN-γ and/or TNF-α antibody. Furthermore, monocytes purified from ET^flox/flox^;Tie2-Cre( + ) mice, which conditionally lack ET-1 in hematopoietic stem cells and vascular endothelial cells, did not produce ET-1 even when stimulated by antigen-specific T cell activation. This study demonstrates the existence of an immune-mediated ET-1 production induced by T cells upon activation through IFN-γ and TNF-α.

## Introduction

A prominent role for the endothelin (ET) system in the physiological regulation of blood pressure has been long recognized^1^. Endothelin 1 (ET-1) is a major factor that induces vasoconstriction and is mainly produced by vascular endothelial cells, the primary source of ET-1 in physiological conditions^1,2^. Binding of ET-1 to the endothelin type A receptor (ETa) results in vasoconstriction, growth, and remodeling effects^1,3^. Focusing on this mechanism, endothelin receptor antagonists were developed and have proven to be among the most effective therapeutic agents, especially for controlling pulmonary hypertension^1,4^.

Recently, in addition to regulation of the circulatory system through ET-1 from vascular endothelial cells, an increasing body of evidence suggests that the ET-1 system may play a role in some experimental models of inflammation^5–11^. Although the ET-1 production system has been well investigated in normal physiology in vascular endothelial cells, whether a specific ET-1 production system works in inflammatory conditions remains to be determined. Some investigators have suggested that immune cells such as macrophages^12–15^ and dendritic cells synthesize ET-1^16^. Production of ET-1 from these cells may occur via lipopolysaccharide stimulation^14^ or an autocrine loop mechanism^16^. However, immune-mediated ET-1 producing system other than LPS-triggering ET-1 production remains to be determined.

For this reason, we postulate the possible relationship between T cells and ET-1 producing cells in an immune-mediated ET-1 production system. In our current study, we explored the existence of an ET-1 production and induction system that is related to the immune system, and demonstrate a newly discovered T cell-mediated vascular endothelial cell-independent ET-1 production system by using human and murine immune cells.

## Results

### ET-1 production by human PBMCs and murine spleen cells following activation of T cells

**Figure 1:**
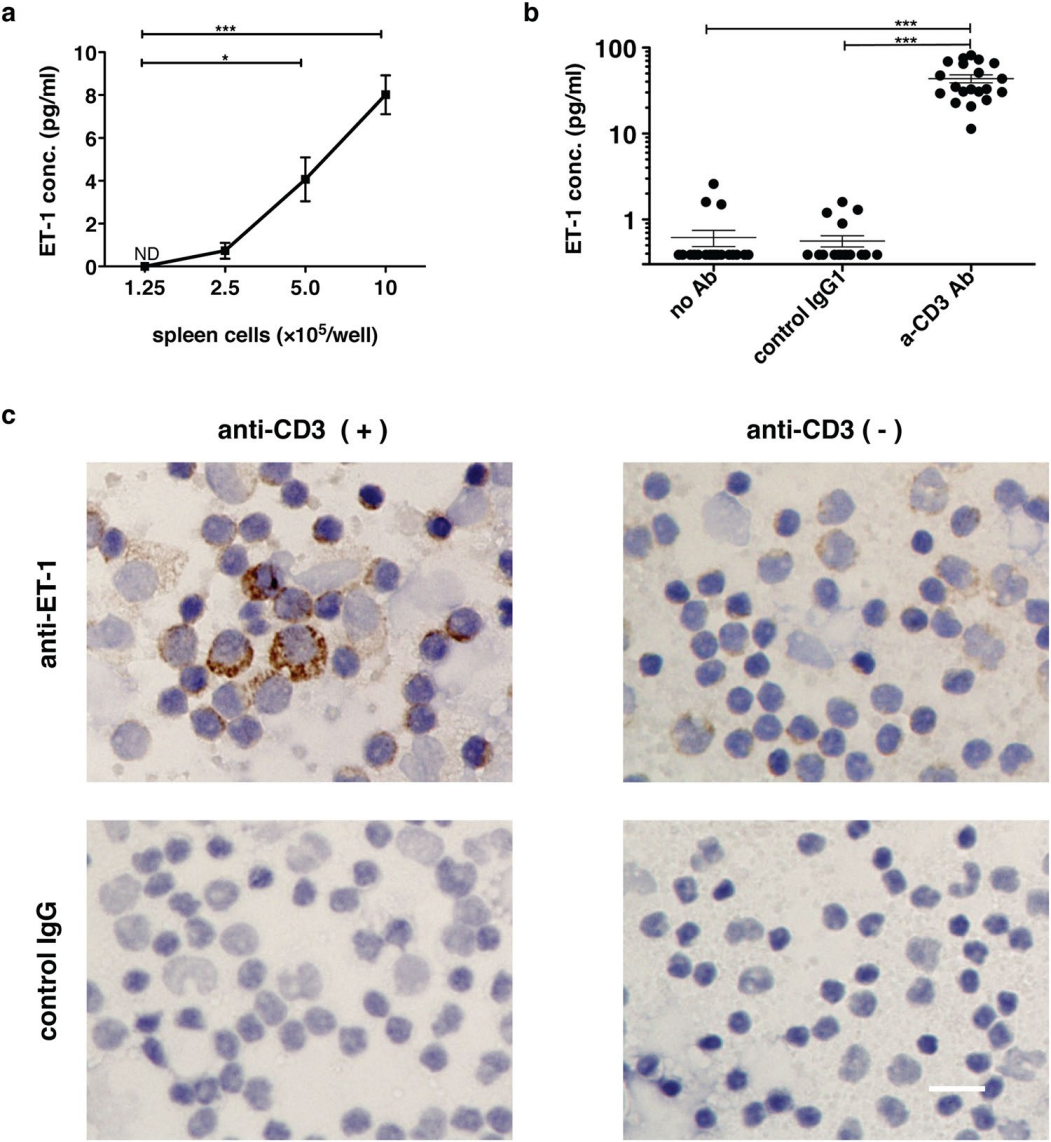
ET-1 production by immune cells following anti-CD3 Ab-mediated activation of T cells in murine spleen cells and human PBMCs. ET-1 production in the culture supernatant of 1 × 10^6^ cells/well of (**a**) murine spleen cells (**b**) human PBMCs from 20 healthy donors cultured for 24 h with or without stimulation with 10 μg/ml immobilized anti-human CD3 Ab or control IgG1. The value under the limit of detection (0.39 pg/ml) with ET-1 ELISA assay was defined as 0.39 pg/ml. The assays were performed in triplicate wells. Data are expressed as the mean ± SEM. *p < 0.05, ***p < 0.001 as compared with negative control by (**a**) one-way ANOVA followed by post-hoc Tukey’s multiple comparison test and (**b**) Kruskal-Wallis followed by post-hoc Dunn’s multiple comparison test. (**c**) Representative images of immunocytochemical staining of human PBMCs with anti-human ET1 mAb. PBMCs were cultured in the presence or absence of immobilized anti-human CD3 Ab for 28 h. Brefeldin A (10 μg/ml) was added to the culture for the last 4 h. After fixation with 4% paraformaldehyde and methanol, DAB staining was performed using anti-hET1 Ab (TR.ET.48.5) or control IgG. Scale bar represents 20 μm. Similar results (**a**) and images (**c**) were obtained in 3 different experiments.

T cells express receptors for some vasoconstriction factors such as angiotensin and 5-hydroxytryptamine^17,18^. Some investigators have used RT-PCR and flow cytometric analysis, and reported that endothelin receptors are expressed in human peripheral T cells^19,20^. Considering that ET-1 may work in an autocrine or paracrine manner, the T cells themselves or adjacent immunocompetent cells could produce ET-1 and stimulate endothelin receptors on activated T cells during inflammation. We investigated whether murine immune spleen cells stimulated with immobilized anti-CD3 Ab can produce ET-1 and observed that such cells produced ET-1 in a cell number-dependent manner (Fig. 1a). Based on this fact, we also tested whether T cell activation could promote PBMCs from 20 healthy donors to produce ET-1. This human *in vitro* study also clearly showed that PBMCs, independent of vascular endothelial cells, produced ET-1 in a manner that was dependent only on the activation of T cells (Fig. 1b). Furthermore, immunocytochemistry showed that cells that produced ET-1 were present among PBMCs activated by immobilized anti-CD3 Ab (Fig. 1c).

### ET-1 production from monocytes induced by activated T cells via IFN-γ and TNF-α

**Figure 2:**
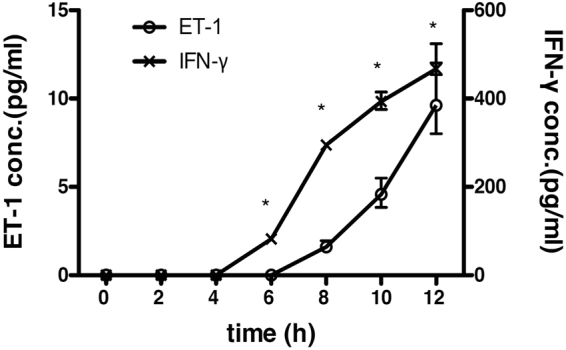
Comparison of the production kinetics between IFN-γ and ET-1 in the culture supernatant of PBMCs stimulated with immobilized anti-CD3 Ab. ET1 production in the culture supernatant of 1 × 10^6^ cells/well of human PBMCs from a healthy donor cultured with 10 μg/ml immobilized anti-human CD3 Ab. The culture supernatants at appropriate times were collected and measured by ET-1 or IFN-γ ELISA. Assays were performed in triplicate wells, and data represent means ± SEM. ND = not detected. ^*^ *P* < 0.001 as calculated compared with negative control (Time 0) by two-way ANOVA followed by post-hoc Sediak’s multiple comparison test. Similar results were obtained in 3 different experiments.

To investigate how ET-1 is produced from PBMCs induced by activated T cells, we compared the production kinetics of IFN-γ and ET-1 from activated T cell-induced PBMCs. The kinetics of ET-1 production was clearly later than that of IFN-γ for activation of T cells, suggesting that T cells may induce other immune cells to produce ET-1 in PBMCs (Fig. 2).

**Figure 3:**
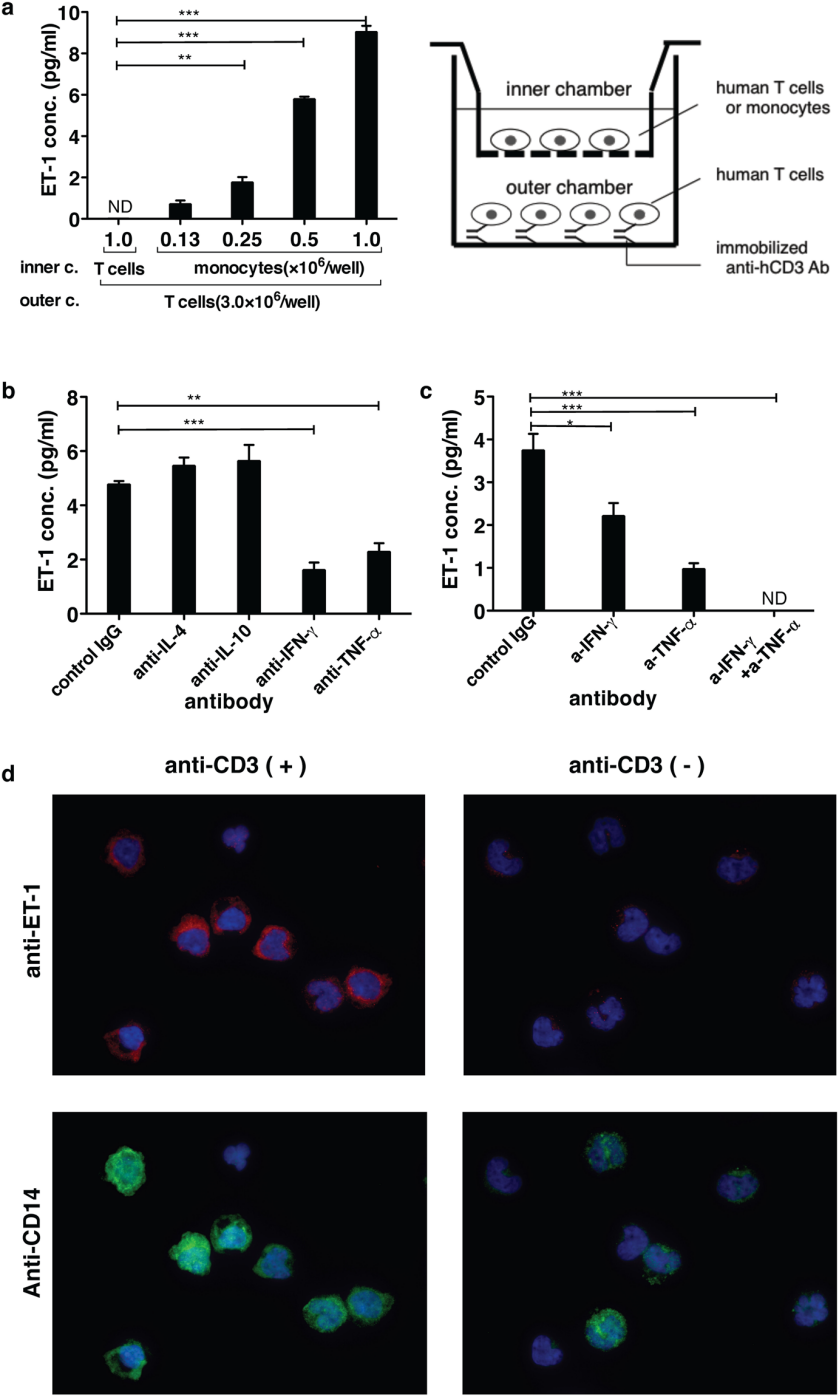
The relationship between T cells and monocytes in ET-1 induction with the double chamber system using human cells. After PBMCs were prepared from 100 ml of whole blood of a volunteer, T cells and monocytes were purified from human PBMCs with Mini-MACS, respectively. Three million T cells were stimulated with immobilized anti-CD3 Ab in the outer chamber of the Transwell system (See the schema on the right of Fig. 3a.). In the inner chamber (**a**), the number of purified T cells or monocytes with magnetic beads is shown on the x axis. For panels (**b**) and (**c**), 1.5 × 10^6^ purified human monocytes were cultured for 24 h. The ET-1 concentration in the supernatant of each culture was measured using ELISA. The concentration of each blocking Ab used was 30 µg/ml. ND = not detected. The assays were performed in triplicate wells. Data are expressed as the mean ± SEM. *p < 0.05, **p < 0.01, ***p < 0.001 as compared with negative control with T cells in the inner chamber (**a**) or positive control (**b**,**c**) by one-way ANOVA followed by post-hoc Tukey’s multiple comparison test. (**d**) Images of the detection of ET-1 production from monocytes purified from PBMCs in the inner chamber. The monocytes in the inner chamber were cultured with T cells in the outer chamber in the presence or absence of immobilized anti-human CD3 Ab for 28 h. Brefeldin A (10 μg/ml) was added to the culture for the last 4 h. After fixation with 4% paraformaldehyde and 0.5% Triton X-100, immunofluorescent staining was performed using anti-ET-1 Ab (TR.ET.48.5) with anti-polyclonal mouse IgG(H + L) conjugated with Alexa Fluor 594 (Red) and anti-human CD14 (1:25 dilution) conjugated with Alexa Fluor 488 (Green). Nuclei were visualized with DAPI (Blue). Similar results and images were obtained in at least 3 different experiments (**a**–**d**).

Based on previous reports showing that ET-1 is synthesized by macrophages and dendritic cells^12–16^, we focused on monocytes as candidates for the source of ET-1 production. T cells and monocytes were purified from PBMCs with magnetic beads and separated with a porous membrane (0.4 μm) using the Transwell system. Purified monocytes in the inner chamber did not produce ET-1 without activated T cells induced by immobilized anti-CD3 Ab in the outer chamber (see Supplementary Fig. S1). Activation of T cells with immobilized anti-CD3 Ab in the outer chamber induced ET-1 production only by monocytes, and not by T cells, in the inner chamber (Fig. 3a). Furthermore, ET-1 production was strongly detected in purified monocytes separated with a porous membrane in the inner chamber by immunofluorescent staining only when T cells were activated with immobilized anti-CD3 Ab in the outer chamber (Fig. 3d). These results indicated that T cells induced monocytes to produce ET-1, possibly via soluble factors released by T cells upon activation. TNF-α is a key cytokine that induces ET-1 production from vascular endothelial cells^21,22^. On the other hands, the cytokines produced by T cells that give rise to macrophages with a phenotype that is distinct from monocytes are IFN-γ, TNF-α, IL-4, and IL-10^23,24^. We next examined the effect of blocking Abs for these cytokines on this T cell-mediated ET-1 production system from monocytes. In the inhibition assay using the double chamber system, Abs against TNF-α and IFN-γ partially inhibited ET-1 production from monocytes, whereas Abs against IL-4 and IL-10 did not (Fig. 3b). Moreover, addition of both Abs blocking IFN-γ and TNF-α completely abolished this ET-1 production from monocytes (Fig. 3c). These findings demonstrated that IFN-γ and TNF-α released from activated T cells induced monocytes to produce ET-1 in human PBMCs.

### ET-1 production from monocytes induced by murine antigen-specific CTL activation

**Figure 4:**
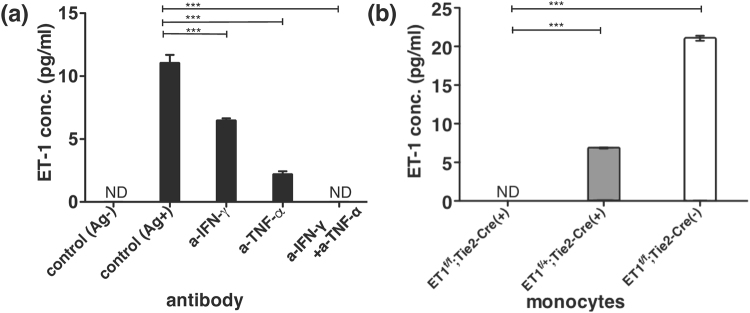
Effect of antigen-specific activation of T cells on monocytes in mice. Three million Tax11-19-specific CTLs were stimulated with 4 × 10^6^ mitomycin C-treated syngeneic spleen cells from HHD mice in the presence or absence of 0.1 μM Tax11-19 peptide as an antigen (Ag) in the outer chamber. In the inner chamber, after bone marrow cells were prepared from 4 mice of femurs in each strain, 1.5 × 10^6^ /well of monocytes purified with Mini-MACS from bone marrow cells of (**a**) HHD mice and (**b**) ET1^flox/flox^;Tie2-Cre(+), ET1^flox/+^;Tie2-Cre(+), and ET1^flox/flox^;Tie2-Cre(−) mice were cultured for 24 h. The concentration of each blocking Ab used was 30 µg/ml. ND = not detected. The assays were performed in triplicate wells. Data are expressed as the mean ± SEM. ***p < 0.001 as compared with positive (**a**) or negative (**b**) control by one-way ANOVA followed by post-hoc Tukey’s multiple comparison test. Similar results were obtained in two experiments (**a**,**b**).

Stimulation by immobilized anti-CD3 Ab induces canonical T-cell receptor signaling, which may not reflect natural T cell activation. Therefore, we next examined whether antigen-specific T cell activation could induce ET-1 production from monocytes in mice. The tax-specific CTL line we developed recognizes a specific antigen, Tax11-19 peptide of HTLV-1 restricted with HLA-A2, upon activation in HLA-A2 transgenic HHD mice^25,26^. Tax-specific CTLs and mitomicyn C-treated syngeneic spleen cells from HHD mice were used as antigen-specific T cells and antigen-presenting cells, respectively. Using magnetic beads, murine monocytes were purified from bone marrow cells in HHD mice. Bone marrow-derived monocytes in the inner chamber produced ET-1 only in the presence of antigen-specific activation of CTLs in the outer chamber (Fig. 4a). Furthermore, ET-1 production from monocytes induced by antigen-specific activation of CTLs was also inhibited by anti-IFN-γ and anti-TNF-α Abs (Fig. 4a). Furthermore, from another point of view, to determine whether monocytes can produce ET-1, we used cells from Tie2-Cre;ET^flox/flox^ mice that allow conditional deletion of ET-1 using the Cre-loxP system^27^. Because Tie2 is expressed in endothelial cells and hematopoietic stem cells^28^, bone marrow-derived monocytes purified from Tie2-Cre( + );ET^flox/flox^ mice should not be able to produce ET-1. Antigen-specific activation of murine CTLs in the outer chamber induced the bone marrow-derived monocytes purified from Tie2-Cre(−);ET^flox/flox^ mice, which do not harbor the Tie2 promotor, to produce ET-1 in the upper chamber. However, ET-1 production was abolished when using cells from Tie2-Cre( + );ET^flox/flox^ mice in the inner chamber (Fig. 4b). All these findings clearly demonstrated that T cells, upon activation in an antigen-specific manner, promoted monocytes to produce ET-1 via the soluble factors of IFN-γ and TNF-α.

## Discussion

ET-1 is a vasoconstriction factor producing powerful contraction of a range of mammalian blood vessel *in vitro*, including human arteries and veins^1^. In physiological condition, ET-1 synthesized in vascular endothelial cells plays a role in regulating vascular tones by affecting vascular smooth muscles. On the other hand, with the implementation of endothelin receptor antagonist in experimental studies, the blockade of ET-1 mediated signaling with endothelin receptor antagonists reduces the level of inflammation in some animal models of LPS-induced lung inflammation^5^, allograft rejection^6^, antigen-induced arthritis^7,8^, antigen-induced allergic rhinitis^10^ and monocrotaline-induced pulmonary artery hypertension^11^. However, how ET-1 is produced and works in such inflammation models other than LPS-induced inflammation remains unknown. Especially in allograft rejection, antigen-induced inflammation and monocrotaline-induced pulmonary artery hypertension models, T cells play a pivotal role in initiating the adaptive immune response^29–32^. In clinical studies, immunosuppressive therapy is effective in some cases of connective tissue disease-related pulmonary artery hypertension (PAH), and ET-1 could play a key role in its pathogenesis^33–36^. In idiopathic and connective tissue disease-associated PAH, the existence of perivascular infiltrates and lymphoid follicles that include adaptive immune cells around pulmonary arteries is a pathological finding of pulmonary hypertension^37–39^. Although these experimental, clinical and pathological studies suggest that the ET-1 production system may be modulated by immune cells, the results are conflicting, and evidence for such actions is largely indirect. Thus, we hypothesized that T cells might induce ET-1 production in immune cells. The present study provides clear evidence regarding a potential role for activated T cells to induce the production of ET-1 as a pressor factor from monocytes in both humans and mice.

Endothelin receptors were expressed in T cells^20^. Because ET-1 is considered to act in an autocrine/paracrine manner, endothelin receptors on T cells could be affected by T cells themselves or adjacent immune cells. ET-1 was not detected in the culture supernatant of immobilized anti-CD3 Ab-stimulated T cells (Fig. 3a), whereas both human PBMCs and murine spleen cells stimulated with immobilized anti-CD3 Ab produced significant levels of ET-1 (Fig. 1a,b). Using immunocytochemistry, we also confirmed the existence of ET-1-producing cells among PBMCs stimulated with immobilized anti-CD3 Ab (Fig. 1c). These results indicated that a vascular endothelial cell-independent ET-1 production system can emerge following activation of T cells in human PBMCs and murine spleen cells. Although the primary source of ET-1 synthesis is regarded as vascular endothelial cells, the peptide is produced by other cell types including epithelial cells in the lung, kidney, and colon^1,40–42^ and the choroid plexus and reactive glial cells in the brain^43,44^. Among immune cells, ET-1 synthesis is detected in macrophages^12–14^ and dendritic cells^16^. However, little is known about the induction mechanism of ET-1 production by immune cells other than the lipopolysaccharides-activation system of macrophages^12^ and the putative autocrine loop mechanism in dendritic cells^16^. To determine how activated T cells could induce immune cells to produce ET-1, we focused on monocytes as a candidate cell type for ET-1-producing immune cells and purified each population of T cells and monocytes with magnetic beads from human PBMCs or murine bone marrow cells. Using the Transwell system, purified T cells, upon activation with immobilized anti-CD3 Ab in the outer chamber, induced monocytes in the inner chamber to produce ET-1 (Fig. 3a, Supplementary Figure S1). Induction of ET-1 production in this noncontact system indicates the existence of soluble mediator molecules between T cells and monocytes. Monocytes/macrophages are highly plastic cells that adopt a variety of activation states. They are broadly divided into classically activated, wound healing, and regulatory macrophages. The important inducers are IFN-γ and TNF-α, IL-4 and IL-10, respectively^23,24^. Assays using Abs that inhibit these cytokines demonstrated that IFN-γ and TNF-α may be key cytokines for directing monocytes to produce ET-1 as well as induction of classically activated macrophages (Fig. 3b,c, Fig. 4a). Furthermore, in addition to artificial stimulation with immobilized anti-CD3 Ab, antigen-specific activation of cytotoxic T cells led to induction of ET-1 production from bone marrow-derived monocytes in mice, and this induction was blocked by anti-IFN-γ and anti-TNF-α Abs in the Transwell system (Fig. 4a). Since IFN-γ and TNF-α are cytokines produced from CTLs or Th1 cells, we suggest that ET-1 is usually produced during Th1 inflammation and adaptive immunity.

Tie2 is a critical molecule in the development of endothelial cells and hematopoietic stem cells^28^. ET1^flox/flox^;Tie2-Cre mice, which harbor the Tie2 promotor, are conditionally deficient in ET-1 production in endothelial cells and immune cells derived from hematopoietic stem cells^27^. The monocytes purified from bone marrow cells in ET1^flox/flox^;Tie2-Cre mice lost the ability to produce ET-1, but this was not seen in ET1^flox/+^;Tie2-Cre and ET1^flox/flox^;Tie2-Cre(−) mice (Fig. 4b). These results also provide evidence for the existence of an ET-1 production system in monocytes induced by T cells upon activation.

The current study suggests that not only acquired immunity may have a potential to directly affect the circulatory system with the newly discovered T cell-mediated ET-1 production mechanism, but also the signal transfer via IFN-γ and TNF-α from T cells to monocytes could play a key role in the regulation of such an immune-mediated ET-1 production system.

In the final part of our study, we focused on the relationship between T cell activation and ET-1 in humans and mice. However, the details of how and where ET-1 is released from monocytes in the immune system are unclear. Further studies will be needed to clarify the physiological and pathological role of the T cell-induced ET-1 production system within the immune system.

## Materials and Methods

### Mice

Transgenic HHD-2 mice (gift from Dr. François Lemonnier, Institute Pasteur, Paris, France) were bred in our colony at the Institute of Experimental Animals at St. Marianna University. HHD-2 mice are characterized by knock-out of the murine β_2_-microglobulin gene, as well as murine H-2D^b^, plus transgenic expression of human HLA-A2.1 with covalently linked human β_2_-microglobulin and a murine D^b^-derived α3 domain to allow interaction with mouse CD8^26^. A tax-specific cytotoxic T lymphocyte (CTL) line was developed from HHD mice. Antigen-specific CTLs recognize the specific antigen of the Tax11-19 peptide of HTLV-1, LLFGYPVYV^25^.

### Generation of tyrosine kinase with immunoglobulin and epidermal growth factor homology domains-2 (Tie2)-Cre( + );ET^flox/flox^ mice

B6.Cg-Edn1 <tm1Ywa> (ET^flox/flox^ transgenic) mice harbor a transgene with loxP sites flanking exon 2 of the ET1 gene^45^. B6.Cg-Tg(Tek-cre)1Ywa (Tie2-Cre transgenic) mice express the Cre recombinase gene under the control of the mouse Tek(Tie2) promoter^46^. Both mouse strains were provided by RIKEN Bioresource Center (Tsukuba, Japan). Tie2-Cre( + );ET^flox/flox^ mice were obtained from crossbreeding these two transgenic lines. To confirm establishment of Tie2-Cre(+);ET^flox/flox^ mice, genomic DNA was prepared from earlobe biopsies from postnatal day 21 mice and used for genotyping with PCR analysis^27^.

### Murine spleen cells and bone marrow cells

All animal studies were approved by the Institute of Experimental Animals Committee at St. Marianna University and the methods in these studies were carried out in accordance with the approved guideline. The murine spleen was removed, the tissue was disrupted, red blood cells were lysed with osmotic lysis, and the single cell suspension was passed through a 70-μm sterile cell strainer. Murine bone marrow cells were obtained from the femur by flushing the shaft with buffer using a syringe with a 25 G needle. After obtaining a single-cell suspension by gently pipetting several times, the cells were passed through a cell strainer to remove cell clumps that may clog the column. Cells were washed with PBS (300 × *g* for 10 minutes) three times.

### Human peripheral blood mononuclear cells (PBMCs)

Peripheral blood samples from 20 healthy volunteers who provided informed and written consent following permission from the institutional review board at St. Marianna University were used for preparation of PBMCs, which were isolated using Lympholyte-H (CEDARLANE, Canada). All methods using PBMCs in human studies were carried out in accordance with the approved guidelines.

### Cell separation

Immune cells including both human and murine total T cells and monocytes were sorted by negative selection on a Mini-MACS with a cell isolation kit (Miltenyi Biotec, Bergisch Gladbach, Germany) according to the manufacturer’s instructions. The efficiency of cell purification was ~95% confirmed by flow-cytometric analysis in each cell purification kit.

### IFN-γ and ET-1 ELISA

IFN-γ and ET-1 in the culture supernatant that was harvested at appropriate times were determined using an ELISA kit (R&D, Minneapolis, MN) according to the manufacturer’s instructions. Values of all samples were expressed as means ± SEM of triplicate measurements.

### Antibodies

Anti-human cytokine blocking Abs were purchased as follows: IFN-γ and tumor necrosis factor-alpha (TNF-α) from eBioscience Inc. (San Diego, CA), interleukin (IL)-4 from U-cytech (Utrecht, Netherland), and IL-10 from Diaclone (Besançon Cedex, France). Anti-murine IFN-γ and TNF-α blocking Abs were purchased from Thermo Scientific (Waltham, MA) and R&D Systems, respectively.

### Immunocytochemistry

ET-1 expression was assessed with immunocytochemistry. PBMCs collected from the culture were prepared on cytospin slides. After fixation with 4% paraformaldehyde and methanol, immunocytochemistry staining was carried out. After blocking with 10% human serum in Block Ace (DS Pharma Biomedical, Osaka, Japan), PBMCs were incubated with diluted primary Abs (anti-ET1 mAb (TR.ET.48.5) 1:200) overnight at 4 °C. Immunodetection was performed using diluted secondary anti-mouse IgG1 Ab conjugated to horseradish peroxidase (1:1,000, Bethyl Laboratories, Montgomery, TX). Visualization was carried out with the Liquid DAB + Substrate Chromogen System (DAKO, Santa Clara, CA). Images were taken using a BZ-9000 fluorescence microscope (KEYENCE, Osaka, Japan).

In the study using Transwell system, after co-cultured with T cells with or without immobilized anti-CD3 Ab in the outer chamber, monocytes collected from the inner chamber were prepared on cytospin slide. After fixation with 4% paraformaldehyde and 0.5% Triton X-100, immunofluorescence was performed using the following antibodies: anti-ET1 mAb (TR.ET.48.5, 1:400 dilution) and Alexa Fluor 594-conjugated anti-mouse IgG(H + L) (1:300 dilution) (Jackson ImmunoReseach Inc., West Grove, PA). Cells were incubated in primary antibody diluted in Can Get Signal immunostain (TOYOBO, Osaka, Japan) at 4 °C overnight. After rinsing thrice with PBS(−), Alexa Fluor 594-conjugated secondary antibody was used for detection. Alexa Fluor 488-conjugated anti-human CD14 (1: 25 dilution) (BioLegend, San Diego, CA) was subsequently used for double staining. After four additional PBS washes, cells were stained with 4′,6-diamidino-2-phenylindole (DAPI) (Sigma-Aldrich, St. Louis, MO). Images were taken using a BZ-9000 fluorescence microscope (KEYENCE, Osaka, Japan).

### Statistical analysis

All of the detection experiments using ELISA were performed in triplicate, and the data were expressed as the mean ± SEM and were analyzed using Graph Pad Prism V.6.0 (San Diego, CA). We used one-way ANOVA followed by *post-hoc* Tukey’s multiple comparison test in Figs 1a, 3a,b, 4a and b. In Fig. 1b, based on the result by D’Agostino & Pearson omnibus normality test, we used Kruskal-Wallis followed by *post-hoc* Dunn’s multiple comparison test. Two-way ANOVA was performed in Fig. 2. Significance was accepted as p < 0.05.

## Electronic supplementary material

Supplementary Figure S1
