# Supplementary material for: T cells upon activation promote endothelin 1 production in monocytes via IFN-γ and TNF-α
Source: Sci Rep. 2017 Nov 3;7:14500. doi: 10.1038/s41598-017-14202-5 (PMC5670167; doi:10.1038/s41598-017-14202-5)
Supplement: Supplementary file 1 — Supplementary Figure S1 [file 41598_2017_14202_MOESM1_ESM.pdf]

Supplementary Information

T cells upon activation promote endothelin 1 production in monocytes via IFN-γ and TNF-α.

Shoshi Shinagawa[1], Takahiro Okazaki[1*], Mari Ikeda[1], Kazuo Yudoh[2], Yaz Y. Kisanuki[3], Masashi Yanagisawa[4], Kimito Kawahata[1] and Shoichi Ozaki[1]

[1] Division of Rheumatology and Allergology, Department of Internal Medicine, St. Marianna University School of Medicine, Kawasaki, Japan

[2] Department of Frontier Medicine, Institute of Medical Science, St. Marianna University School of Medicine, Kawasaki, Japan

[3] Department of Neurology, Division of Neurogenetics, The Ohio State University Wexner Medical Center, Columbus, Ohio State, USA

[4] International Institute for Integrative Sleep Medicine (WPI-IIIS), University of Tsukuba, Tsukuba, Japan

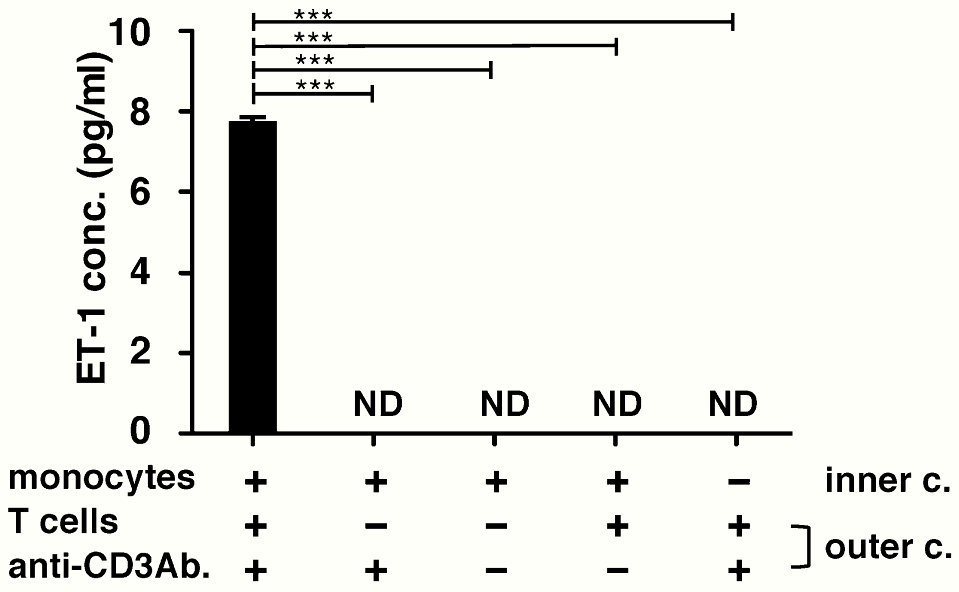

Figure S1: Monocytes do not produce ET-1 without activation of T cells induced by immobilized anti-hCD3 Ab in the Transwell system.

After PBMCs were prepared from 100 ml of whole blood of a volunteer, T cells and monocytes were purified from human PBMCs with Mini-MACS, respectively. Three million of T cells were stimulated with or without immobilized anti-CD3 Ab in the outer chamber of the Transwell system (See the schema on the right of Figure 3a.). In the inner chamber, one million of purified human monocytes were cultured for 24 h. The ET-1 concentration in the supernatant of each culture was measured using ELISA. ND = not detected. The assays were performed in triplicate wells. Data are expressed as the mean ± SEM. The ND value under the limit of detection (0.39 pg/ml) with ET-1 ELISA assay was calculated as 0.39 pg/ml. The assays were performed in triplicate wells. *** $p < 0.001$ as compared with activated T cells-induced ET-1 production from monocytes as positive control by one-way ANOVA followed by post-hoc Tukey's multiple comparison test. Similar results were obtained twice.
